# Measuring empathic, person-centred communication in primary care nurses: validity and reliability of the Consultation and Relational Empathy (CARE) Measure

**DOI:** 10.1186/s12875-015-0374-y

**Published:** 2015-10-23

**Authors:** Annemieke P. Bikker, Bridie Fitzpatrick, Douglas Murphy, Stewart W. Mercer

**Affiliations:** General Practice and Primary Care, Institute of Health and Wellbeing, University of Glasgow, 1 Horselethill Road, Glasgow, G12 9LX UK; School of Medicine, University of Dundee, Mackenzie Building, Kirsty Semple Way, Dundee, DD2 4BF UK

**Keywords:** Empathy, CARE Measure, Practice nurses, Validation, Reliability, Primary care

## Abstract

**Background:**

Empathic patient-centred care is central to high quality health encounters. The Consultation and Relational Empathy (CARE) Measure is a patient-rated experience measure of the interpersonal quality of healthcare encounters. The measure has been extensively validated and is widely used by doctors in primary care but has not been validated in nursing. This study assessed the validity and reliability of the CARE Measure in routine nurse consultations in primary care.

**Methods:**

Seventeen nurses from nine general medical practices located in three Scottish Health Boards participated in the study. Consecutive patients (aged 16 years or older) were asked to self-complete a questionnaire containing the CARE Measure immediately after their clinical encounter with the nurse. Statistical analysis included Spearman’s correlation and principal component analysis (construct validity), Cronbach’s alpha (internal consistency), and Generalisability theory (inter-rater reliability).

**Results:**

A total of 774 patients (327 male and 447 female) completed the questionnaire. Almost three out of four patients (73 %) felt that the CARE Measure items were very important to their current consultation. The number of ‘not applicable’ responses and missing values were low overall (5.7 and 1.6 % respectively). The mean CARE Measure score in the consultations was 45.9 and 48 % achieved the maximum possible score of 50. CARE Measure scores correlated in predicted ways with overall satisfaction and patient enablement in support of convergent and divergent validity. Factor analysis found that the CARE Measure items loaded highly onto a single factor. The measure showed high internal consistency (Cronbach’s alpha coefficient = 0.97) and acceptable inter-rater reliability (*G* = 0.6 with 60 patients ratings per nurse). The scores were not affected by patients’ age, gender, self-perceived overall health, living arrangements, employment status or language spoken at home.

**Conclusions:**

The CARE Measure has high face and construct validity, and internal reliability in nurse consultations in primary care. Its ability to discriminate between nurses is sufficient for educational and quality improvement purposes.

## Background

Patients consistently score empathy and the human aspects of care as top priorities in their health care [1–4]. Research has linked empathic care to higher levels of patient satisfaction [5–7], enablement [8–10] and improved health outcomes [8, 10–14]. Its importance is emphasised in healthcare policies [15–17] and professional codes of conduct [18, 19]. Healthcare practitioners are increasingly expected to demonstrate their interpersonal skills in terms of empathic, patient-centred care in practice and training [20, 21]. Measurement is crucial to evaluate this aspect of quality of care and to obtain feedback on individual practitioners.

The Consultation and Relational Empathy (CARE) Measure is a patient-assessed measure of the quality of the encounter with healthcare professionals [3, 21]. Ten items ask patients’ perception of the practitioner’s ‘relational empathy’, defined as the healthcare practitioner’s ability to:

- understand the patient’s situation, perspective and feelings (and their attached meanings);
- communicate that understanding and check its accuracy, and
- act on that understanding with the patient in a helpful (therapeutic) way [3, 21].

The development of the measure was based on a review of existing measures and qualitative interviews with patients, and their feedback on the individual items in order to create a measure that was meaningful regardless of the patients’ socioeconomic status [21]. We did this by assessing the views of patients living in areas of high or low socioeconomic deprivation and, in an iterative process, developed, validated and tested the CARE Measure in primary care consultations with general practitioners (GPs) [21, 22].

Since its development and validation with general practitioners (GPs) in the UK [21], the measure has been extensively validated with a range of physician groups in primary and secondary care [22–26]. It has been widely used nationally (including in GP appraisal and revalidation) and internationally, and has been translated and validated in various languages [23, 24, 27]. However, to date nurses have not been included in this expanding body of work on the CARE Measure. Given the increasing role of nurses in primary care in many countries, it would seem timely to asses whether the CARE Measure is valid and reliable in this professional group. It would be scientifically wrong to assume that a measure developed primarily for use with GPs will also be valid and reliable with nurses. The role of nurses in primary care is distinct from that of GPs; in the United Kingdom (UK), practice nurses are employed by GPs to carry out routine annual reviews of a limited number of single chronic disease, and some also do minor illness clinics. GPs, on the other hand, deal with a wide range of clinical issues, including the management of most mental health problems and patients with complex multimorbidity of chronic diseases.

In carrying out the current study we have a number of hypotheses to be tested based on our previous work on empathy and the CARE Measure:

1. We would expect the CARE measure to be relevant to most consultations with practice nurses, as we have found for primary care and secondary care doctors [22, 25, 26].
2. Since the CARE measure reflects patients’ views on generic interpersonal skills, we would expect it to be valid and reliable in primary care nurses, similar to what we have found for GPs and other doctor groups [22, 25, 26].
3. We would expect the CARE measure to load onto a single factor in factor analysis as found in other studies [22, 24–26].
4. As in this previous work [22, 24–26], we would predict the CARE Measure would show convergent validity with patient satisfaction but divergent validity with patient enablement, since the latter is a construct quite distinct from satisfaction [28].
5. We would also predict that the CARE Measure would be related to factors such as consultation length and continuity, as shown in our previous work with doctors [22, 25, 26].

## Methods

### Sampling, data collection and ethics

Ten out of 55 randomly selected GP Practices within a 40 mile radius of the study office responded positively to an invitation to participate in the study. The Practices represented three Scottish Health Board areas and combined provided the opportunity to collect data on the consultations of 20 nurses (19 practice nurses and 1 nurse practitioner). The aim was to collect self-completed patient questionnaires for 50 consecutive consultations with each participating nurse. The sampling strategy ensured that patients from a range of socioeconomic levels would be included in the study, but we did not have sufficient funding to specifically sample from high and low deprivation areas as we did in our original validation work [21, 22]. The consultation number was based on previous work that demonstrated the required sample size to effectively discriminate between GPs [22].

Practice receptionists gave consecutive adult patients (16 years or older) a questionnaire when they checked in for their appointment with a participating nurse. This is the same approach that we have used in our previous validation studies with GPs [21, 22]. Patients completed the questionnaire immediately after the consultation and placed it in a sealed box in the waiting room. The questionnaire contained:

- The 10 item CARE Measure [21]
- A question on the importance of the CARE Measure items to their consultation (rated on a 4 point scale from 1 = not important to 4 = very important) which we have used previously [22].
- An overall satisfaction question (rated on a Likert scale from 1 = completely satisfied to 7 = completely dissatisfied). This was included because perceived empathy is known to be an important determinant of patient satisfaction and thus would be predicted to correlate positively with CARE measure scores, and thus provide evidence of convergent validity [5–7].
- The six items contained in the Patient Enablement Instrument (PEI) [28]. The PEI was included because although enablement is related to satisfaction and CARE measure scores, it is a different construct. It would be predicted to correlate less strongly with the CARE measure than patient satisfaction, and thus provide evidence of divergent validity.
- Questions we have used previously [12, 22, 28] on relational continuity (how well the patients knows the nurse, rated on a Likert scale from 1 = don’t know at all to 5 = know very well), whether or not previously seen by nurse, consultation length, satisfaction with consultation length (from 1 = very poor to 6 = excellent)
- Socio-demographic details (self-perceived overall health, age, gender, living arrangements, employment status and language spoken at home).

Data were collected between September 2012 and October 2013.

### Scoring of the CARE Measure

The 10 CARE Measure items are rated on a 5-item response scale from 1 = poor to 5 = excellent. The overall score is the sum of the ten items with 10 being the lowest possible score and 50 the highest. Up to two not applicable (N/A) responses or missing values are allowed and these are replaced by the average item score.

As we wanted to directly compare the findings of the current study with our previous work with GPs [21, 22] we did not attempt to ‘weight’ the scores.

### Ethics

Ethical approval was obtained from the National Research Ethics Service (NRES) Committee North West, Preston (Reference 12/NW0607, on 2/8/2012). All nurses who participated in the study provided written informed consent. Informed consent was not required from the patients who decided to complete the questionnaire, because the questionnaire was anonymous and did not ask for identifiable information. Completion of the questionnaire was voluntary and the optional nature of the study was explained in the information on the front of the questionnaire.

### Analysis

The data were analysed using SPSS (version 21) and urGENOVA software via its associated wrapper program GS4 for the reliability analyses [29, 30] as was done in our previous studies [25, 26]. Descriptive methods were used to describe the sample, calculate the CARE Measure and PEI scores, and check the variability in the data. As data distributions were skewed, differences between groups were assessed through non-parametric tests. The perceived relevance and face validity of the CARE Measure was assessed by analysing the number of not applicable and missing values for each of its 10 items as well as the patients’ rating of the importance of the 10 items, as in previous studies [22–27]. Construct validity was examined through factor analysis (principal component analysis with varimax rotation and Kaiser normalisation) and correlations (Spearman’ rho) between the CARE Measure items, and PEI and the overall satisfaction measure. The internal reliability of the CARE Measure was assessed through Cronbach’s alpa. The ability of the measure to discriminate between nurses was assessed using Generalisability-theory (G-Theory) and associated Decision D studies [22, 25, 26].

## Results

One practice with three nurses withdrew from the study, thus 17 nurses took part. Completed questionnaires were obtained for 774 practice nurse consultations (37–55 per nurse). Consultations included appointments for clinics for chronic disease management such as diabetes, coronary heart disease and chronic obstructive pulmonary disease. One nurse provided acute care only. The age of patients attending these consultations ranged from 16 to 93 years (mean age = 54.9 years, SD = 18.2), 447 patients (57.8 %) were female, and 391 patients (51 %) rated their overall health as good or very good (Table 1).

**Table 1:** Demographic data of participating patients

	Sample size (*n*)	% of total sample
Gender		
Male	290	37.5
Female	447	57.8
Missing values	37	4.8
Age group		
16–29 years	87	11.2
30–44 years	105	13.6
45–65 years	298	38.5
>65 years	234	30.2
Missing values	50	6.5
Overall Health Status		
Very good/ good	394	50.9
Fair	239	30.9
Bad/ very bad	103	13.4
Missing values	38	4.9
Living arrangements		
With Partner/Spouse	453	58.5
Not with Partner/Spouse	281	36.3
Missing Values	40	5.2
Language Spoken at Home		
English	729	94.2
Other	6	0.8
Missing Values	39	5.0
Employment status		
Employed (full- or part-time, including self-employed)	279	36.0
Unemployed (looking for work)	41	5.3
Unfit to work	92	11.9
Retired	265	34.2
Looking after home/family	19	2.4
In education	19	2.5
Other	18	2.1
Missing	41	5.3
Help with Questionnaire		
Yes	64	8.3
No	681	88.0
Missing Values	29	3.7

In terms of consultation characteristics, the length of consultations ranged from 1 to 50 min (mean consultation length = 13 min, SD = 7.6 min). Around three-quarter of patients (76 %) reported a previous consultation with the nurse and half the patients (50 %) reported that they knew the practice nurse quite well or very well. CARE Measure scores were weakly correlated with how well patients knew the nurse (Spearman’s rho 218, *p* = 0.000) and with consultation length (rho 0.123, *p* = 0.001).

### Relevance to current consultation

Overall, almost three-quarters of patients (73 %) perceived the CARE Measure items as very important to their current consultation. Older patients and patients with worse self-reported overall health tended to score the importance of the items higher than younger patients (*p* = 0.000) and patients with better health status (*p* = 0.033) (Table 2). No statistical differences were found for the other patient characteristics.

**Table 2:** Patients’ perceived importance of the CARE Measure items to their consultation

	Little or No Importance (%)	Moderate Importance (%)	Very Important (%)	*p*- value
All Consultations	39 (5.1)	137 (17.7)	562 (72.6)	
Age group				0.000
29	12 (14.1)	26 (30.6)	47 (56.3)	
30–44	7 (6.8)	28 (27.2)	68 (66.0)	
45–65	12 (4.1)	43 (14.7)	237 (81.2)	
> 65	5 (2.2)	33 (14.8)	185 (83.05)	
Gender				ns
Male	20 (7.1)	40 (14.3)	220 (78.6)	
Female	17 (3.9)	93 (21.4)	325 (74.7)	
Overall Health Status				0.033
Very good/good	22 (5.7)	86 (22.5)	275 (71.8)	
Fair	10 (4.3)	38 (16.5)	182 (79.1)	
Bad/very bad	5 (5.0)	10 (9.9)	86 (85.1)	
Living arrangements				ns
With Partner/Spouse	23 (5.3)	76 (17.4)	338 (77.3)	
Not with Partner/Spouse	12 (4.5)	52 (19.4)	204 (76.1	
Language Spoken at Home				ns
English	37 (5.2)	132 (18.6)	539 (76.1)	
Other	0 (0)	1 (16.7)	6 (83.3)	
Employment Status				0.081
Employed (full- or part-time, including self-employed)	18 (6.8)	56 (21.1)	192 (72.2)	
Unemployed (looking for work)	5 (12.5)	8 (20.0)	27 (67.5)	
Unfit to work	5 (5.7)	12 (13.8)	70 (80.5)	
Retired	5 (2.0)	41 (16.1)	208 (81.9)	
Looking after home/family	1 (5.9)	3 (17.6)	13 (76.5)	
In education	1 (5.0)	6 (30.0)	13 (65.0)	
Other	1 (6.3)	1 (6.3)	14 (87.5)	
Help with Questionnaire				ns
Yes	2 (3.2)	6 (9.5)	55 (87.3)	
No	35 (5.4)	125 (19.2)	492 (75.5)	

‘Not applicable’ responses in the CARE Measure amounted to only 6 % of the total possible ‘not applicable’ responses (444/7740 items; see Table 3). For CARE Measure items 1 to 8, the average number of not applicable responses was 3 % (*n* = 181), ranging from 0.6 % for item 1 to 5 % for item 2. The highest number of “not applicable” responses were recorded for items 9 (*n* = 112, 15 %) and 10 (*n* = 151, 20 %), which relate to ‘taking control’ and ‘making a plan of action’ respectively. The total number of responses for which there was missing data was 124, representing less than 2 % of the total possible number of missing responses (i.e. 124/7740). The missing responses were evenly distributed across the CARE Measure items.

**Table 3:** Applicability and missing values by CARE Measure items

CARE Measure item	Not Applicable responses (%)	Missing values (%)
item 1 Making you feel at ease	5 (0.6)	11 (1.4)
item 2 Letting you tell your story	42 (5.4)	11 (1.4)
item 3 Really listening	16 (2.1)	12 (1.6)
item 4 Being interested in you as a whole person	14 (1.8)	13 (1.7)
item 5 Fully understand your concerns	41 (5.3)	13 (1.7)
item 6 Showing care and compassion	15 (1.9)	12 (1.6)
item 7 Being positive	22 (2.8)	15 (1.9)
item 8 Explain things clearly	26 (3.4)	11 (1.4)
item 9 Helping you to take control	112 (14.5)	12 (1.6)
item 10 Making a plan of action with you	151 (19.5)	14 (1.8)
Total	444 (5.7)	124 (1.6)

Three or more ‘not applicable’ or missing responses were given by 76 (9.8 %) patients. Patients with worse self-reported health had less occurrences of three or more ‘not applicable’ or missing responses than patients with better self-reported health status (*χ*^2^ = 5.860, *p* = 0.53). No statistical associations were found for the other patient characteristics (results not shown).

### Performance of the CARE Measure

The overall mean CARE Measure score for all practice nurses was 45.9 (SD 5.9) and the mean CARE Measure scores per practice nurse ranged from 42.6 to 47.9 (*p* = 0.005). Individual patient scores ranged from 20 (minimum possible score being 10) to the maximum score of 50. Nearly half of practice nurse consultations (48 %) received the maximum possible score. The distribution of the scores showed a skew of −1.5 and a kurtosis of 1.8.

Explanatory factor analysis on the CARE Measure items, PEI and satisfaction questions showed three factors with the CARE Measure items on one factor with high loadings (0.883-0.967), indicating a robust internal structure of the CARE Measure (Table 4). The PEI items loaded on the second factor, and the satisfaction measures on the third factor. The three factors explained 83 % of the variance.

**Table 4:** Factor analysis of the CARE Measure, PEI, and satisfaction items

	Factor 1	Factor 2	Factor 3
1. Making you feel at ease	.889	.185	.028
2. Letting you tell your story	.954	.127	.019
3. Really listening	.968	.145	.006
4. Being interested in you as a whole person	.966	.148	.007
5. Fully understand your concerns	.967	.118	.014
6. Showing care and compassion	.967	.144	.008
7. Being positive	.883	.102	.008
8. Explain things clearly	.912	.134	−.009
9. Helping you to take control	.937	.036	−.006
10. Making a plan of action with you	.890	.007	.001
PEI 1 Ability to cope with life	−.182	.794	−.065
PEI 2 Ability to understand illness	−.234	.844	−.029
PEI 3 Ability to cope with illness	−.214	.885	−.001
PEI 4 Ability to keep self health	−.217	.890	.051
PEI 5 Confidence about health	−.196	.869	.052
PEI 6 Ability to help self	−.208	.887	.038
Overall satisfaction	−.039	−.015	.786
Rating consultation time	.039	.061	−.781

Correlations between the CARE Measure scores and patient enablement and overall satisfaction supported construct (convergent) validity in relation to overall satisfaction (Spearman’s rho 0.54, *p* = 0.000), and as expected less (divergent) with patient enablement (Spearman’s rho 0.19, *p* = 0.000).

The CARE Measure showed weak but significant positive relationships with consultation length (Spearman’s rho 0.12, *p* = 0.002) and how well patients knew the nurse (Spearman’s rho 0.22, *p* = 0.000). The patient characteristics of age, gender, self-perceived overall health, living arrangements, employment status and language spoken at home were unrelated to the CARE Measure score.

In terms of the CARE Measures ability to discriminate between nurses a moderate level of agreement (inter-rater reliability) between patients was found with 50–60 patient ratings per nurse (Table 5). As expected, the reliability of the measure increased with the number of completed questionnaires per practice nurse.

**Table 5:** Reliability of the CARE Measure in differentiating between nurses in relation to number of questionnaires

Number of Completed per Practice Nurse	Reliability(G-Theory Analysis)
37	0.479
50	0.554
60	0.598
100	0.713

The measure showed high internal reliability (Cronbach’s alpha coefficient 0.97). Removal of any item weakened this internal reliability (results not shown).

## Discussion

This study aimed to determine the reliability (internal and inter-rater) and validity (face and construct) of the CARE Measure as an outcome measure of routine primary care nursing consultations. The results suggested that overall the patients viewed the CARE Measure items as highly relevant to their consultations with nearly three quarters rating the items as ‘very important’ to their consultation. The low number of ‘not applicable’ responses and missing values in the CARE Measure items further suggests that the CARE Measure is relevant to primary care nursing consultations and supports the face validity of the measure.

Construct validity of the CARE Measure was demonstrated by a moderate/strong correlation with overall satisfaction (convergent validity) and a weaker correlation with patient enablement (divergent validity). The factor analysis further supported the construct validity as the CARE Measure items loaded highly on one factor showing that they capture the same concept, which was different from overall satisfaction and patient enablement.

In the present study mean CARE Measure scores per nurse were generally high and this restriction in range, caused by the ceiling effect of high scores within the studied cohort of nurses, limited variation between nurses. Despite this phenomenon, the measure could still effectively discriminate between the nurses with 60 questionnaires per nurse with an acceptably high level of stability (*G* = 0.6). While this inter-patient reliability is short of the commonly accepted level of 0.8 for a stand-alone ‘high stakes’ assessment [31], the results are consistent with other tools, such as Objective Structured Clinical Examinations (OSCE), commonly used to inform summative decisions in other contexts [32]. For ‘low stakes’ assessments, the CARE Measure is likely to be useful even at much lower numbers of patients per nurse. Furthermore, a larger number of nurses in a more diverse range of practices may have yielded more variation between nurses and thus higher G scores, and further research on a larger and more diverse sample may be useful to determine this. The Cronbachs alpha coefficient of 0.97 indicated high internal reliability. Moreover, the mean CARE Measure scores were not affected by any of the measured patients’ characteristics and this gives some confidence that the measure can be used in different primary care nursing settings.

### Relationship to literature

Overall, the mean CARE Measure score for all practice nurses (45.9) was somewhat higher than in the primary care setting with GPs (40.9) [22]. Additionally, the primary care nurses required a slightly higher number (60) of completed CARE Measures than the GPs for whom 50 CARE Measure scores were sufficient to estimate reliably the mean CARE Measure score for an individual GP [22]. The reason for the higher number of required patient questionnaires for practice nurses is that the practice nurses had less variability in the data. The high level of perceived importance of the CARE Measure items to everyday practice nurse consultations in this study (73 %) was similar to those with general practitioners (76 %) [22]. In the latter, it was also found that older patients and patient with worse health status (in terms of long-standing illness, psycho-social or emotional problems) tended to rate the items as more relevant. The high perceived relevance of the measure to clinical encounters has also been found in similar studies outside primary care [25, 26] and in international settings [24, 27]. The finding that most “not applicable” responses were given to item 9 (helping you to take control) and item 10 (making a plan of action with you) was also in agreement with previous studies [22–26]. As the majority of consultations were routine nursing appointments it could be the case that within that context empowerment and shared-decision making were perceived as less relevant by some patients. Finally, the significant but weak positive associations between estimated consultation length, how well patients know the nurse and CARE Measure scores were similar to those shown in previous studies [22, 25]

### Strengths and weaknesses

A strength of the study was that it builds on earlier work on the CARE Measure and adds to the body of knowledge on its reliability and validity across healthcare disciplines. It was also a reasonably large number of patients, though the number of nurses was lower than we had hoped for. The study had some limitations. First, the participating nurses were volunteers and the study may have been open to sampling bias. This could have led to the high consultation scores and limited the range in performance and the resulting reliability of the inventory. Secondly, one practice withdrew from the study (due to circumstances unrelated to the study) and this reduced the number of participating nurses from 20 to 17. A larger sample size, as employed in previous studies [22, 25, 26], may have captured a wider potential spectrum of possible population performance among nurses and may have found it easier to discriminate between different nurses and demonstrate higher levels of reliability. Another limitation was that it was underestimated how many patients would not return the questionnaires. This resulted in some nurses having collected less than 50 questionnaires even though more than 50 questionnaires had been handed out to patients.

### Implications for practice and future studies

As the CARE Measure was originally developed and validated in primary care with GPs, the findings of this study suggest that the CARE Measure can be used reliably with primary care practice nurses as well. Practice nurses working in general practice tend to have serial consultations with patients allowing them to establish ongoing relationships with them. In this study, most patients felt that they knew the nurse quite well or very well. Further work is required across nursing to establish if the CARE Measure is also reliable, valid, acceptable and feasible in other nursing settings, such as secondary care or acute care community clinics such as sexual health, and also in allied healthcare professionals such as physiotherapists, occupational therapists, podiatrists, and so on.

Although capturing patients’ views on health professionals’ interpersonal skills is now widely regarded as an important feature of high quality health care systems, the evidence that such feedback in itself leads to change in professionals consulting behaviour (and thus improves scores) is equivocal [33]. In order to support healthcare practitioners to improve or maintain their CARE Measure scores and/or the ability to provide an empathic service, earlier work developed [34, 35] and piloted [36] the CARE Approach framework. This inter-disciplinary resource is derived from the CARE Measure and wider literature and covers the four interactive components of Connecting, Assessing, Responding and Empowering with the aim of fostering empathy and patient-centredness in clinical encounters [34, 35]. The use of the CARE Measure as well as the CARE Approach feeds into current healthcare policies and professional codes of conduct on maintaining, enhancing and monitoring empathic, person-centred care [15–19].

## Conclusion

Research shows that an emphatic, person-centred approach to care is linked with improved experiences of care, higher patient enablement and better health outcomes. The CARE Measure appears relevant to, valid and reliable in routine practice nurse consultations. Completed CARE Measures from 60 patient consultations are required to provide a stable enough view on which to base feedback for educational and quality improvement purposes in relation to relational empathy. As patients’ demographics did not affect the CARE Measure scores, it can be used in different primary care settings. In conclusion, the results underpin the CARE Measure as a useful tool to facilitate the patients’ voice in providing feedback to practice nurses on their relational empathy.
